# Esophageal cancer practice guidelines 2017 edited by the Japan esophageal society: part 2

**DOI:** 10.1007/s10388-018-0642-8

**Published:** 2018-08-31

**Authors:** Yuko Kitagawa, Takashi Uno, Tsuneo Oyama, Ken Kato, Hiroyuki Kato, Hirofumi Kawakubo, Osamu Kawamura, Motoyasu Kusano, Hiroyuki Kuwano, Hiroya Takeuchi, Yasushi Toh, Yuichiro Doki, Yoshio Naomoto, Kenji Nemoto, Eisuke Booka, Hisahiro Matsubara, Tatsuya Miyazaki, Manabu Muto, Akio Yanagisawa, Masahiro Yoshida

**Affiliations:** 1 Department of Surgery, Keio University School of Medicine, 35 Shinanomachi, Shinjuku-ku, Tokyo, 160-8582 Japan; 2 Department of Radiology, Graduate School of Medicine, Chiba University, Chiba, Japan; 3 Department of Gastroenterology, Saku Central Hospital, Nagano, Japan; 4 Gastrointestinal Medical Oncology Division, National Cancer Center Hospital, Tokyo, Japan; 5 Department of Gastrointestinal Tract Surgery, Fukushima Medical University School of Medicine, Fukushima, Japan; 6 Department of Endoscopy and Endoscopic Surgery, Gunma University Hospital, Maebashi, Gunma Japan; 7 Department of General Surgical Science, Gunma University Graduate School of Medicine, Maebashi, Gunma Japan; 8 Department of Surgery, Hamamatsu University School of Medicine, Hamamatsu, Shizuoka Japan; 9 Department of Gastroenterological Surgery, National Kyushu Cancer Center, Fukuoka, Japan; 10 Department of Gastroenterological Surgery, Osaka University Graduate School of Medicine, Suita, Osaka Japan; 11 Department of General Surgery, Kawasaki Medical School, Okayama, Japan; 12 Department of Radiation Oncology, Yamagata University School of Medicine, Yonezawa, Japan; 13 Department of Frontier Surgery, Graduate School of Medicine, Chiba University, Chiba, Japan; 14 Department of Clinical Oncology, Kyoto University Hospital, Kyoto, Japan; 15 Department of Pathology, Kyoto Prefectural University of Medicine, Kyoto, Japan; 16 Department of Hemodialysis and Surgery, Chemotherapy Research Institute, International University of Health and Welfare, Ichikawa, Japan

**Keywords:** Practice guidelines, Esophagus, Cancer

## Endoscopic treatment

### Summary

Endoscopic resection (ER) includes endoscopic mucosal resection (EMR), wherein the affected mucosal lesion is held or aspirated and resected with a snare, and endoscopic submucosal dissection (ESD), which refers to en bloc resection of an extensive lesion using an IT knife or hook knife [1–4]. Other endoscopic treatments available include photodynamic therapy (PDT), argon plasma coagulation (APC), and electromagnetic coagulation therapy.

### General remarks

#### Indications for endoscopic resection

Among lesions in which the depth of invasion does not extend beyond the mucosal layer (T1a), those confined within the mucosal EP or the LPM are only extremely rarely associated with lymph-node metastasis; therefore, endoscopic resection is a sufficiently radical treatment for these lesions. Lesions extending up to the muscularis mucosae or slightly infiltrating the submucosa (up to 200 μm) are also amenable to mucosal resection; however, they are associated with an elevated risk of lymph-node metastasis. Therefore, these represent relative indications [5, 6]. Furthermore, about 50% of the lesions that show deeper (more than 200 μm) invasion into the submucosa (T1b) are associated with metastasis, and in such cases, even if they are classified as superficial carcinomas, should be treated in the same manner as advanced carcinomas. Mucosal resection covering 3/4 of the entire circumference is likely to be associated with postoperative cicatricial stenosis. Therefore, sufficient explanation should be given to the patient prior to the operation and preventive measures must be taken [7, 8].

#### Diagnosis by histopathology of the resected tissue specimens

There are limitations to all the modes of diagnosis of the depth of tumor invasion prior to treatment. It is also difficult to accurately determine the depth of invasion of extensive lesions. Furthermore, preoperative diagnosis of the histologic type of the invasive tumors or that of vascular invasion is impracticable. Histopathologic examination of the resected tissue specimens is, therefore, important for determining whether an additional treatment is required or not, and diagnosis of tissue specimens obtained by en bloc resection is indispensable.

#### Treatment of lesions not amenable to endoscopic resection

Insufficient elevation of the mucosa after submucosal injection may pose difficulty in additional ER of residual marginal lesions after ER, or ER after radiotherapy or chemoradiotherapy. These cases and cases with a bleeding tendency are not suitable for ER, and other treatment options such as PDT [9] and APC would need to be considered.

#### Superiority of en bloc resection

En bloc resection is desirable for histologic diagnosis of the resected specimens. ESD enables en bloc resection of lesions that were formerly subjected to fractional resection. Further development of equipment and spread of improved techniques are anticipated.

#### Complications

Various complications, including bleeding (0.2%), esophageal perforation (1.9%), and post-resection cicatricial stenosis (6.0–16.7%), have been reported in association with ER [10]. Sufficient explanation should be provided to the patients, and measures must be taken for prevention/treatment of these complications.

#### CQ18: is additional treatment recommended in cases detected to have pT1a-MM lesion following endoscopic treatment for superficial esophageal cancer?

### Recommendation statement

There is strong evidence to recommend an additional treatment in patients identified as having a pT1a-MM lesion with positive vascular invasion after endoscopic treatment (rate of consensus: 85% [17/20]; strength of evidence: D).

### Explanatory note

There are no reports of randomized comparative or case–control studies demonstrating the usefulness of additional treatment in patients in whom the resected specimens collected at endoscopic treatment are histopathologically diagnosed as pT1a-MM lesions.

According to the reports based on the results of surgical treatment, the frequency of lymph-node metastasis in resected specimens obtained from patients with pT1a-MM squamous cell carcinoma was 0–27%, and summarization and analysis of data from major reports revealed that it was present in 30/210 cases (14.2%; 95% CI 9.85–19.76) [11–18]. There are few or no reports on the frequency of metastasis in pT1a-MM adenocarcinoma cases, however, that in cases of pT1a adenocarcinomas is reported to be in the range of 0–5%; summarization and analysis of data from major reports revealed that it was presented in 91/1882 cases (4.9%; 95% CI 3.95–5.9) [19–21]. Meanwhile, the frequency of recurrent lymph-node metastasis in cases diagnosed from the resected specimens collected at endoscopic treatment as pT1a-MM disease was 0–4.2% for squamous cell carcinoma, with a tallied frequency of 5/223 (2.24%; 95% CI 0.73–5.15) [5, 17, 22], and 0% for adenocarcinoma [23]. For squamous cell carcinomas, in particular, the frequency of lymph-node metastasis differed markedly between cases with pT1a-MM disease identified in surgical specimens and that identified in endoscopically resected specimens. This difference in the frequency of lymph-node metastasis is considered to be mainly attributable to the difference in the method of histopathologic diagnosis between surgical specimens and endoscopically resected specimens. As surgical specimens are larger in size as compared to endoscopically resected specimens, the possibility of cases diagnosed as pT1a-MM disease of including pT1b cases cannot be ruled out. As a ground for this presumption, it has been reported that the frequency of lymphatic invasion in pT1a-MM cases substantially differs between cases who have undergone endoscopic resection and those who have undergone surgery (pT1a-MM in endoscopically resected cases: 0–8.1% [5, 17]; surgically treated cases: 18.2–41.2% [11–14, 17]).

Reports of studies conducted to identify the risk factors for lymph-node metastasis in cases with superficial cancer of the esophagus limited to pT1a-MM cases are scarce. It was from the analysis of the data of 50 pT1a-MM cases in one study, that the frequency of lymph-node metastasis significantly differed between lymphatic invasion-negative cases and lymphatic invasion-positive cases (negative cases: 4/38 (10.5%); positive cases: 5/12 (41.7%) [14]. Multivariate analysis to identify the risk factors for lymph-node metastasis revealed an odds ratio for positive lymphatic invasion of 3.63–6.11 for T1 cases overall [15, 16], 3.83 for pT1a-MM/pT1b-SM1 cases [14], and 7.333 when the analysis was limited to pT1a-MM cases [17]. Assessment of the risk factors for metachronous metastasis in cases treated by endoscopic resection revealed a frequency of lymph-node or distant metastasis of 3.73% (15/402) for the pT1 cases overall, 0.36% (1/280) for pT1a-EP/LPM cases, 4.29% (3/70) for pT1a–MM cases, 11.7% (2/17) for pT1b-SM1 cases, and 25.7% (9/35) for pT1b–SM2 cases; hence, the frequency increased progressively with advancing depth of invasion, and multivariate analysis identified depth of invasion as the sole significant risk factor, with a hazard ratio of 13.1 (95% CI 1.3–133.7, *p* = 0.03) for pT1a-MM vs. pT1a-EP/LPM [22]. For superficial carcinomas overall, on the other hand, positive lymphatic invasion failed to be identified as a significant risk factor for metachronous metastasis; however, when the analysis was limited to only pT1a cases, the 5-year cumulative incidence of metastasis was significantly higher in the lymphatic metastasis-positive cases as compared to the lymphatic metastasis-negative cases (46.7% vs. 0.7%, *p* < 0.0001) [22]. Cases diagnosed as having pT1a-MM cases after endoscopic resection had a greater risk of recurrence of metastasis as compared to those diagnosed as having pT1a-EP/LPM disease and positive lymphatic invasion may be cited as a risk factor, although it is difficult to arrive at a conclusion, because all the papers reviewed represented retrospectively accumulated case reports and an additional treatment mainly consisting of chemoradiotherapy was administered to lymphatic invasion-positive cases among the patients treated by endoscopic resection.

Surgical treatment or chemoradiotherapy is considered as a radical additional treatment in patients diagnosed by histopathology of the endoscopically resected specimens as having pT1a-MM disease. Gratifying therapeutic results in surgically treated T1a patients have been reported, with a reported 5-year disease-specific survival rate of 98–100% and overall survival rate 82–100% [17, 18, 20]. Meanwhile, the mortality rate from postoperative complications has been reported to be in the range of 0.2–3.6% [14, 18, 21, 24]. In regard to the results of chemoradiotherapy for cStage I (cT1N0M0) disease, the reported 4-year overall survival rate was 80.5%, 5-year overall survival rate was 66.4%, and 5-year disease-specific survival rate was 76.8%, despite the inclusion of a significant proportion of cT1b cases (85.2% for cT1a cases) [25, 26]. Esophageal fistula (3.2%), esophagostenosis (3.2%), Grade 3 cardiac ischemia (1%), and respiratory failure (2.8%) were reported as serious late complications, but there has been no report of treatment-related death [25, 26]. In patients given additional chemoradiotherapy after endoscopic resection, the 5-year overall survival rate and disease-specific survival time were both 100% among pT1a-MM cases as well as T1b-SM1 cases, and the 3-year survival rate was 92.9% for pT1a-MM cases, although the sample sizes in the studies were small; neither report contained any detailed description on adverse events, although there were no cases of serious adverse events or treatment-related death [5, 27]. Taking into consideration the benefit–risk balance, the additional treatment may be useful for patients diagnosed by histopathology of the endoscopically resected specimens as having pT1a-MM disease, who are at a high risk of recurrence.

From the above results, the strength of evidence was rated as D, considering that most of the reports cited represented retrospective case accumulations, and no recommendation based on high-level evidence has been made yet. Chemoradiotherapy, which is the mainly adopted modality for additional treatment, is covered by the national health insurance. Taking into account the benefit–risk balance, strength of evidence, and patient preferences, we conclude that there is strong evidence to recommend additional treatment in patients identified as having a pT1a-MM lesion with positive vascular invasion after endoscopic treatment.

## Surgical treatment

### Surgery for cervical esophageal carcinoma

#### Summary

In the treatment of cervical esophageal carcinoma, simultaneous laryngectomy is often required; therefore, preoperative chemoradiotherapy or definitive chemoradiotherapy is often undertaken in an attempt to conserve the larynx. Larynx-preserving surgery enables conservation of vocal function, although it is associated with an increased risk of aspiration and pneumonia, necessitating the need for caution while selecting this treatment. Lowering of the QOL due to the loss of vocal function poses a serious problem in patients who have undergone combined laryngectomy. No significant difference in the post-treatment prognosis has been reported so fact between cervical esophageal carcinoma patients treated by surgery and radical chemoradiotherapy. The appropriate treatment in these patients should be selected with due consideration given to the QOL, etc.

#### General remarks

Since cervical esophageal carcinoma develops in a region densely packed with important structures such as the trachea, large blood vessels, nerves, and the thyroid, it is frequently associated with malignant invasions of the adjacent organs. Lymph-node metastasis is also frequently encountered; therefore, it is not uncommon for the malignancy to be at an advanced stage at diagnosis. There are a significant number of cases in which surgery is indicated inasmuch as widespread metastasis is uncommon, unlike the case in thoracic esophageal cancer. A major problem in surgery for cervical esophageal cancer is that simultaneous laryngectomy is also indicated in many cases. Under these circumstances, surgery may be performed after tumor shrinkage is obtained by preoperative chemoradiotherapy in an effort to preserve the larynx, or radical chemoradiotherapy may be administered, followed by salvage surgery in the event of detection of residual disease or recurrence.

Larynx-preserving surgery is indicated for patients in whom the tumor has not invaded the pharynx, larynx, or trachea. Conservation of vocal function is the utmost benefit of this option, although it is associated with the risk of aspiration or pneumonia; not uncommonly, primary tracheotomy is required. Therefore, sufficient consideration should be given as to the indication and choice of operative procedure, e.g., an additional aspiration-preventive measure such as laryngeal elevation could be employed.

Combined laryngectomy (laryngopharyngoesophagectomy) is indicated for patients with tumors invading the pharynx, larynx, and trachea. The procedure may even be indicated for patients without direct pharyngeal invasion, in whom sufficient preservation of the esophagus to perform anastomosis with intestinal graft is difficult. Marked lowering of QOL due to loss of vocal function poses a serious problem in patients who have undergone combined laryngectomy.

Reconstruction after surgical resection of cervical esophageal carcinoma is frequently performed using a free jejunal graft [28] or a gastric tube [29]. The method of first choice is reconstruction using a free jejunal graft, although reconstruction using a gastric tube is chosen for cases in which the disorder is complicated by thoracic esophageal cancer or in which the cervical esophageal cancer extends caudad to involve the thoracic esophagus.

The frequency of lymph-node metastasis in cases of cervical esophageal cancer is relatively high, although it is confined in most cases to the cervical region and a part of the upper mediastinum; therefore, lymph-node dissection is primarily targeted at lymph nodes in these regions. Nevertheless, reports on the outcomes of lymphadenectomy in patients with cervical esophageal cancer are few as yet, and further investigation is needed.

No significant difference in the post-treatment prognosis has been reported until date between cervical esophageal carcinoma patients treated by surgery alone and those treated by radical chemoradiotherapy. Selection among the available treatment options should be made with due consideration given to the post-treatment QOL, etc.

## Surgery for thoracic esophageal carcinoma

### Summary

Thoracic esophageal carcinoma is often accompanied by extensive lymph-node metastasis in the cervical, thoracic, and abdominal regions. Therefore, it is a common practice that, in T1b-SM 2, 3 or more advanced cases regarded as advanced cancer cases, a right thoracotomy with esophagectomy and lymphadenectomy of the cervical, mediastinal, and upper abdominal regions is carried out. According to the revision of the Japanese Classification of Esophageal Cancer, supraclavicular lymph nodes [#104] are classified in Group 2, to ensure 3-fields’ lymphadenectomy for D2 resection in the surgical treatment of middle thoracic esophageal carcinoma.

In thoracoscopic surgery, thoracic manipulations are currently also carried out with the patient in the prone position, whilst, previously, thoracic manipulations were predominantly undertaken with the patient in the left-lateral decubitus position. This is still at the stage of clinical research. A randomized comparative study to compare the long-term outcomes of this type of surgery vs. conventional standard surgery with thoracotomy has been started (JCOG1409 Study), and the results are awaited.

### General remarks

Thoracic esophageal carcinoma is frequently associated with extensive lymph-node metastasis in the cervical, mediastinal, and upper abdominal regions. Therefore, it is common practice to perform a right thoracotomy to meet the need for adequate dissection of the mediastinal lymph nodes, along with esophagectomy and lymphadenectomy in lymph-node stations of the cervical, thoracic and abdominal regions to complete the entire extent of resection. Depth of invasion beyond T1a-MM is a predictor of lymph-node metastasis, and stage T1b-SM 2, 3 lesions should be counted as advanced carcinomas [17, 30].

The extent of lymph-node dissection should be determined according to individual cases after preoperative evaluation of the location, size, and depth of invasion of the main lesion by imaging modalities such as CT, ultrasonography (US), magnetic resonance imaging (MRI), and PET, because the distribution and incidence of lymph-node metastasis vary with the aforementioned parameters. Based on the analysis of data from a nationwide registry conducted by the Japan Esophageal Society [31], the supraclavicular lymph nodes [#104] are placed in Group 2, to ensure three fields’ lymph-node dissection for D2 dissection in the treatment of middle thoracic esophageal carcinoma in the 11th edition of the Japanese Classification of Esophageal Cancer. It is not feasible to dissect the supraclavicular lymph nodes [#104] via thoracic manipulation, and a cervical approach is necessary for secure lymph-node dissection in this region.

It is common practice that radical surgery for thoracic esophageal carcinoma is usually accomplished using a combination of three approaches: the cervical, thoracic, and abdominal approaches. The mediastinal approach has also been proposed as an alternative to the cervical approach for dissection of the cervical paraesophageal lymph nodes [#101].

Thoracoscopy-assisted esophagectomy with esophageal reconstruction has been reported as promising surgical procedures, in view of its minimal invasiveness, radical curability, and favorable long-term outcomes, although studies are ongoing. Various procedures such as endoscopy/laparoscopy-assisted esophagectomy with esophageal reconstruction and mediastinoscopy- or laparoscopy-assisted transhiatal esophagectomy (blunt resection of the esophagus) have been reported, and analysis of the reported cases during the 2011–2013 period in the National Clinical Database (NCD) revealed that 37.6% of the patients underwent endoscopy/laparoscopy-assisted surgery, which was reported as a safe approach with a mortality rate of 2.44%, against an overall mortality rate of 3.03%. The indications for this approach vary among institutions; it has been adopted even for cT3 cases at some institutions, and in patients who have received preoperative chemoradiotherapy at other institutions.

Some techniques have been introduced for ensuring safe endoscopic surgery with a reduced operation time and improved accuracy of lymph-node dissection, including direct manipulations through a small incision via a minor thoracotomy, video-assisted thoracoscopic surgery (VATS) with minor thoracotomy, and hand-assisted laparoscopic surgery (HALS) involving manipulation with one hand inserted into the abdomen. While thoracic manipulations have been predominantly carried out with the patient in the left-lateral decubitus position, complete endoscopic thoracic manipulations have been increasingly performed with the patient placed in the prone position in recent years. Mediastinal lymph-node dissection using a mediastinoscope inserted via a cervical incision and laparoscopic transhiatal lymph-node dissection are some of the other procedures described. Reports have suggested that endoscopy/laparoscopy-assisted surgery enables conservation of the vasculature and nerves while confirming the microanatomy, and also increases the accuracy of lymph-node dissection, as it allows higher power visualization. A randomized comparative study to assess the long-term outcomes of this type of surgery as compared to the conventional standard surgery with a thoracotomy has been initiated (JCOG1409 Study), and the results are awaited [32].

## Surgery for carcinoma of the esophagogastric junction (abdominal esophageal carcinoma)

### Summary

There is no unanimity of opinions as to treatment policy and surgical procedures for carcinoma of the esophagogastric junction, particularly adenocarcinoma according to Nishi’s classification or Siewert type II carcinoma. Based on a retrospective analysis, the Japanese Gastric Cancer Association—Japan Esophageal Society Joint Working Group proposed the optimal extent of lymph-node resection for esophagogastric junction carcinomas measuring ≤4 cm in diameter. Prospective clinical studies to determine the optimal extent of lymph-node resection for more advanced tumors are currently in progress.

### General remarks

For definition of carcinoma of the esophagogastric junction, Siewert’s classification is used overseas, whereas, in Japan, Nishi’s classification is adopted by both the Japanese Gastric Cancer Association and the Japan Esophageal Society. In Siewert’s classification, type I lesions are often handled as carcinomas of the thoracic esophagus and type III lesions as cardiac carcinomas. Squamous cell carcinomas in Nishi’s classification, on the other hand, are often treated as thoracic esophageal cancers. Opinions are still divided as to treatment policy and surgical procedures for adenocarcinomas in Nishi’s classification and Siewert type II carcinoma.

Carcinoma of the esophagogastric junction may be associated with extremely extensive lymph-node metastasis involving the cervical region, mediastinum, upper abdomen, and areas circumjacent to the abdominal aorta, and no unified view has been reached in regard to the appropriate extent of lymph-node dissection. The Japanese Gastric Cancer Association—Japan Esophageal Society Joint Working Group has laid down recommendations in respect of the extent of lymphadenectomy on the grounds of the dissection effect index (rate of metastasis × 5-year survival rate of patients with metastasis) derived from retrospective analysis of data from surgically treated cases. The efficacy of lymphadenectomy in accordance with this scheme is expected to be verified by future accumulation of cases. Nevertheless, the problems with retrospective analysis of tumors is that the patients are confined to those with tumors measuring ≤4 cm in diameter and that the subject population includes only a small number of cases with dissection of the lymph nodes in the upper and middle mediastinal regions and areas circumjacent to the abdominal aorta. A prospective clinical study to evaluate the outcomes depending on the extent of lymphadenectomy for more advanced tumors is currently in progress.

The Japanese Gastric Cancer Association—Japan Esophageal Society Joint Working Group has proposed a definition of the esophagogastric junction based on endoscopic findings. In the algorithm used as a guide for the extent of lymph-node dissection, as well, lesions are defined according to the principal location of the center of the tumor, i.e., whether it is located proximal or distal to the junction. In the clinical practice setting, however, the junction can scarcely be identified by endoscopy in cases of advanced carcinoma, and that frequent, concurrent hiatal herniation interferes with positional estimation of the junction even by fluoroscopic exploration or CT. Thus, it may be said that only but an obscure judgment about the location of the junction can be obtained in the clinical setting. The extent of resection of the esophagus and stomach is determined in accordance with the extent of lymph-node dissection, and the range of operative procedures available extend from total esophagogastrectomy to lower third esophagectomy plus proximal gastrectomy. In surgery for carcinoma of the esophagogastric junction, the surgical invasiveness is affected not only by the extent of resection, but also by the surgical approach; therefore, the treatment selection must be approached by taking into consideration the balance between the surgical invasiveness and curability of the adopted procedure.

## Perioperative management and clinical path

### Summary

Various improvements have been made to the clinical path system for esophageal cancer at facilities overseas and in Japan, in an effort to implement safe perioperative management with a reduced incidence of complications; however, convincing evidence is still to be presented. The clinical significance of the new concept of perioperative management introduced in recent years; namely, Enhanced Recovery after Surgery (ERAS) or fast-track surgery in surgical resection of the esophagus has drawn increasing attention.

### General remarks

A clinical path is a standard medical practice plan containing information on the patient’s condition, goals of medical practice, and relevant evaluations and records, and represents a procedure for improving the quality of medical care through analysis of deviations from the standard. With the introduction of the Diagnosis-Related Group/Prospective Payment System (DRG/PPS) in the 1980s, clinical paths aimed primarily at shortening the length of hospitalization and reducing the medical fees were introduced [33]. In Japan, introduction of clinical paths for many disorders began in the 1990s concurrently with the introduction of the Diagnosis Procedure Combination (DPC) system. Clinical paths are generally thought to be important for promoting patient-centered collaborative (team) medical care, including perfection of informed consent as well as for improving the quality of medical care and education of personnel.

Various improvements have been made in the clinical path system for esophageal cancer at facilities overseas and in Japan, in an effort to implement safe perioperative management, with a reduced incidence of complications. It has generally been recognized that preparation of a simple clinical path for esophageal cancer entails greater difficulty as compared to that for carcinomas of other digestive organs, because of the diversity and interinstitutional inequity of procedures and perioperative management techniques, and because of individual differences in the reaction to invasiveness. An increasing number of facilities have been introducing a clinical path for esophageal cancer for safe perioperative management, in parallel with the introduction of minimally invasive operations including endoscopy-aided surgery; however, convincing evidence demonstrating its clinical usefulness is still awaited [34, 35].

In recent years, the new concept of Enhanced Recovery after Surgery (ERAS) or fast-track surgery has been introduced for perioperative management in Europe and the United States. The ERAS Group organized in 2001 under the European Society for Clinical Nutrition and Metabolism (ESPEN) published an ERAS protocol for colectomy in 2004 [36], which has since been applied to perioperative management for various surgeries. Fast-track surgery is a multimodal rehabilitation program with integrated introduction of evidence-based procedures as an approach to patient care to expedite recovery after surgery. Currently, this term is essentially synonymous with ERAS. The Clinical significance of ERAS and fast-track surgery in cases of esophagectomy has recently been investigated, with the results indicating reductions in the incidence of complications, duration of hospitalization, and mortality rate, even though the level of evidence is still not high at present [37–40].

Perioperative management of patients with esophageal cancer has, heretofore, been assessed by comparative evaluation of the usefulness of clinical paths established at individual facilities from their independent standpoints. From now on, however, the clinical significance of ERAS/fast-track surgery as perioperative management procedures needs to be verified.

## Chemotherapy for unresectable advanced/recurrent esophageal cancer

### Summary

Chemotherapy is used as the only systemic therapy modality under various settings in the treatment of esophageal cancer. Chemoradiotherapy and preoperative chemotherapy are used for cStage I–Stage IV local esophageal cancer, and chemotherapy is also used for unresectable advanced/recurrent esophageal cancer. Combination therapy with cisplatin + 5-FU is used for unresectable advanced/recurrent esophageal cancer, although there is no clear evidence of its ability to prolong the survival. Taxanes and other drugs are used as the second-line therapy in patients who become refractory to the first-line therapies, but these have only been reported in phase II studies involving a small number of patients, and should be used carefully.

### General remarks

Systemic chemotherapy is used as the standard therapy for unresectable advanced/recurrent esophageal cancer. Although no comparative study with untreated controls has clearly demonstrated the ability of chemotherapy alone to prolong the survival, both the efficacy of monotherapy and combination therapy has been reported, and chemotherapy is used as standard therapy.

### Drugs and drug combinations that have been shown to be effective as the first-line therapy

Monotherapy with 5-FU, platinum drugs, taxanes, vinca alkaloids, etc., has been reported to be associated with a response rate of 15–40% and a median survival duration of approximately 3–10 months. Combination therapy has been shown to be associated with even higher response rates (20–60%) than monotherapy (Table 1) [41–43]. Many studies have reported the efficacies of combination therapy with 2 or 3 drugs, whereas only one study has compared the efficacy of combination therapy versus monotherapy. Most of these studies were phase II studies involving a small number of patients. As two-drug combination therapies, the combination of cisplatin and 5-FU, which are expected to have a synergistic effect, and the combination of nedaplatin and 5-FU are used. Combined therapy with cisplatin + 5-FU is considered to be the standard therapy for patients with unresectable advanced/recurrent esophageal cancer. A three-drug combination therapy, a taxane given in combination with cisplatin + 5-FU, has been shown to be highly effective, with a reported response rate of 60% [44, 45], but it is unknown whether this therapy can prolong the survival. Therefore, at present, it is considered desirable to use this three-drug combination therapy in clinical studies. A comparative study of combined cisplatin + 5-FU therapy and 2-weekly docetaxel combined with cisplatin + 5-FU is currently ongoing (JCOG1314 Study), and the results of the study are awaited.

**Table 1:** Reports of the first-line therapy for unresectable advanced/recurrent esophageal cancer

Regimen	*N*	Response rate (%)	Progression-free survival (month)	Median survival (month)
Cisplatin 100 mg/m^2^ on day 1 5-FU 1000 mg/m^2^/day on days 1–5/every 3 weeks	SCC 44	35	6.2	7.6
Cisplatin 70 mg/m^2^ on day 1 5-FU 700 mg/m^2^/day on days 1–5/every 3 weeks	SCC 39	35.9	Patients with response 3.5	Patients with response 9.5
Nedaplatin 90 mg/m^2^ on day 1 5-FU 800 mg/m^2^/day on days 1–5/every 4 weeks	SCC 42	39.5	2.5	8.8
Doxorubicin 30 mg/m^2^/day on day 1 5-FU 700 mg/m^2^/day on days 1–5 Cisplatin 14 mg/m^2^/day on days 1–5/every 4 weeks	SCC 41	43.9	5.0	10.1
Docetaxel 30 to 40 mg/m^2^/day on days 1 and 15 5-FU 800 mg/m^2^/day on days 1–5 Cisplatin 80 mg/m^2^ on day 1/every 4 weeks	SCC 55	62	5.8	11.1

*SCC* squamous cell carcinoma

### Drugs and combination therapies shown to be effective as the second-line therapy

In regard to the second-line therapy for patients who become refractory to cisplatin + 5-FU, no drugs have been clearly shown to prolong the survival. Drugs that are likely to show efficacy other than fluoropyrimidines and platinum drugs should be used, but the benefit–harm (toxicity) balance should be carefully considered (Table 2). Monotherapy with taxanes, such as docetaxel and paclitaxel, is often used [46, 47]. The significance of readministration of drugs used in the first-line therapy and combination therapy [48] for these patients has not been established.

**Table 2:** Reports of second- or subsequent-line therapy for unresectable advanced/recurrent esophageal cancer

Regimen	*N*	Response rate (%)	Progression-free survival (month)	Median survival (month)
Docetaxel 70 mg/m^2^ every 3 weeks	SCC 46^a^ AC 3 Others 2	20	2.3	8.1
Paclitaxel 100 mg/m^2^ on days 1, 8, 15, 22, 29, and 35/every 7 weeks	SCC 52	44.2	3.9	10.4
Docetaxel 30 mg/m^2^/day on day 1 Nedaplatin 50 mg/m^2^ on day 1/every 2 weeks	SCC 48	27.1	3.1	5.9
Nivolumab 3 mg/kg/every 2 weeks	SCC 64	17	1.5	10.8

*SCC* squamous cell carcinoma, *AC* adenocarcinoma

^a^Including 14 patients with the initial treatment

Although there have been a few reports on molecular-targeted drugs, epidermal growth factor receptor (EGFR) inhibitors have been reported to be associated with response rates in the range of 10–20%. A comparative study of an EGFR inhibitor gefitinib and placebo in patients receiving the second-line therapy for esophageal cancer, including adenocarcinoma, failed to demonstrate any usefulness of gefitinib [49]. In the future, development of biomarkers, etc., may allow the usefulness of EGFR inhibitors to be demonstrated in particular subsets of subjects, but, at present, their usefulness in the treatment of esophageal cancer remains unknown.

### Drugs and combination therapies shown to be effective as the third-line therapy

For patients who become refractory or intolerant to the first- and second-line therapies, no drugs have been demonstrated to be effective, and palliative symptomatic treatment is recommended. A phase II study reported the efficacy of nivolumab, an immune checkpoint inhibitor with a new mechanism of action [50], but a phase III comparative study is required to validate its applicability in clinical practice.

## Radiotherapy

### Summary

For definitive radiotherapy, concurrent chemoradiotherapy is recommended. The potential usefulness of preoperative chemoradiotherapy for resectable advanced cancer is being investigated in an ongoing clinical study. Chemoradiotherapy or radiotherapy alone is indicated for unresectable patients according to the PS. Palliative radiotherapy is considered for cStage IVb esophageal cancer patients presenting with obstruction. A total dose of 60 or 50.4 Gy is often prescribed for chemoradiotherapy, and it is considered that unnecessary prolongation of the treatment duration should be avoided.

### General remarks

Randomized comparative studies and their meta-analyses have demonstrated that concurrent chemoradiotherapy is more effective than radiotherapy alone for definitive treatment of esophageal cancer [51, 52]. Therefore, concurrent chemoradiotherapy is considered preferable, unless its use is precluded by factors such as advanced age, presence of complications, or any other reasons.

Radiotherapy is indicated for patients with residual lesions in the local or regional lymph nodes. An additional (chemo-) radiotherapy is considered when there is residual cancer after endoscopic treatment for T1a or T1b cancer, or when the patient is suspected to have lymph-node metastasis.

Preoperative chemotherapy is the standard treatments for resectable advanced cancer in Japan. Patients who are not suitable for surgery or who do not wish to undergo surgery are given definitive chemoradiotherapy. In addition, preoperative chemoradiotherapy for these patients is being investigated in an ongoing clinical study. Chemoradiotherapy is indicated for unresectable cancer patients with a good PS, and subsequently, surgery may be considered. Radiotherapy alone may be considered for patients with a poor PS. Palliative radiotherapy may be considered for cStage IVb esophageal cancer patients presenting with obstruction. Radiotherapy may be used not only in patients with postoperative residual lesions and untreated patients, but also in those with postoperative recurrence without distant metastasis.

At present, in most facilities, CT-based three-dimensional treatment planning is performed, which allows optimization of the doses to the tumor and risk organs, enabling highly accurate treatment. When radiotherapy alone is performed, since the local control rate may decrease due to accelerated repopulation of the tumor cells, it is considered that unnecessary prolongation of the treatment duration should be avoided [53]. In regard to the optimal total dose for definitive treatment, a randomized comparative study of chemoradiotherapy at a total dose of 50.4 Gy versus 64.8 Gy, conducted mainly by the US Radiation Therapy Oncology Group (RTOG), failed to demonstrate the superiority of the higher dose [54]. In Japan, chemoradiotherapy mainly using 60 Gy has been reported, but clinical studies using 50.4 Gy have also been reported, expecting reduction of the late toxicities of chemoradiotherapy and salvage surgery after definitive radiation. In clinical practice, the dose should be determined considering the several factors such as the patient’s general condition, tumor volume, irradiation area, and doses to the risk organs. When radiotherapy alone is performed, a total dose of 60–70 Gy is commonly prescribed.

## Multidisciplinary treatment

### Preoperative/postoperative adjuvant therapy

#### Summary

At present, the standard treatment for cStage II and III thoracic esophageal cancer in Japan is preoperative chemotherapy with cisplatin + 5-FU, followed by surgery. On the other hand, in Europe and North America, the standard treatment is preoperative chemoradiotherapy followed by surgery. A randomized comparative study to confirm the superiority of preoperative docetaxel + cisplatin + 5-FU (DCF) therapy and that of preoperative chemoradiotherapy (cisplatin + 5-FU, radiotherapy at 41.4 Gy) over the currently used preoperative regimen of cisplatin + 5-FU (JCOG1109 Study) is ongoing.

#### General remarks

In recent years, multidisciplinary treatment, including chemotherapy, radiotherapy, and surgery, has been used for esophageal cancer. The JCOG9204 Study conducted in Japan compared the outcomes of surgery alone with those of surgery plus postoperative chemotherapy with cisplatin and 5-FU [55]. While no significant difference in the overall survival were observed between the two groups, the 5-year disease-free survival (DFS) was significantly better in the surgery plus postoperative chemotherapy group (55%) than in the surgery alone group (45%); furthermore, this improved prognosis was particularly evident in the pathological lymph-node metastasis-positive cases. As a result, surgery plus postoperative chemotherapy became the standard treatment in Japan for patients with histopathologically diagnosed lymph-node metastasis after surgical resection. Subsequently, the JCOG9907 Study investigated the optimal timing, in relation to surgery, of adjuvant chemotherapy with cisplatin + 5-FU, and showed that 5-year overall survival was significantly better in the preoperative chemotherapy plus surgery group (55%) than in the surgery plus postoperative chemotherapy group (43%) [56]. Thereafter, preoperative adjuvant chemotherapy with cisplatin + 5-FU followed by radical surgery came to be adopted as the standard of care for cStage II and III thoracic esophageal cancer patients in Japan.

On the other hand, in Europe and North America, preoperative chemoradiotherapy followed by radical surgery is used as the standard treatment. Preoperative chemoradiotherapy yields a higher local control rate than preoperative chemotherapy alone, but may also increase the risk of perioperative complications and surgery-related mortality. So far, in Japan, local control is achieved by accurate lymph-node dissection during surgery, and preoperative radiotherapy has been thought to be unnecessary. In Europe and North America, several randomized comparative studies investigating the usefulness of preoperative chemoradiotherapy have been reported [57], because adequate local control has not yet been achieved by surgery. The CROSS trial, which is a large-scale randomized comparative study conducted in the Netherlands, showed that the overall survival was significantly longer in the preoperative chemoradiotherapy + surgery group than in the surgery alone group (median overall survival, 49.4 vs. 24.0 months) [58]. On the other hand, there were no significant differences in the incidence of postoperative complications between the two groups.

The results of a subgroup analysis in the JCOG9907 Study suggested that the additive effect of the currently used preoperative chemotherapy with cisplatin + 5-FU may be insufficient for improving the prognosis in patients with cStage III thoracic esophageal cancer, and that either preoperative chemotherapy with a more intensive regimen or preoperative chemoradiotherapy may need to be attempted in the future, aimed at better local control. Taxane antitumor agents (paclitaxel/docetaxel) are thought to be effective in patients with esophageal cancer. Recently, DCF therapy, in which docetaxel is added to cisplatin + 5-FU therapy has attracted attention. The JCOG1109 Study, which was started in 2012, is a randomized comparative study performed to confirm the superiority of preoperative DCF therapy and that of preoperative chemoradiotherapy (cisplatin + 5-FU, radiotherapy at 41.4 Gy) over the currently used preoperative regimen of cisplatin + 5-FU, and the results of the study are awaited, so that the standard treatment for cStage II and III thoracic esophageal cancer can be established in Japan [59].

## Chemoradiotherapy

### Summary

Chemoradiotherapy has been demonstrated to yield a greater prolongation of the survival than radiotherapy alone in patients with locally advanced esophageal cancer. It is considered as the standard of care in non-surgical treatment, and chemoradiotherapy aimed at complete cure is indicated for cStage 0 to IVa cancer. Although a study comparing chemoradiotherapy and surgery alone in resectable cases reported that chemoradiotherapy can be expected to have equivalent efficacy to surgery, no studies have directly compared the two, and it is speculated that the standard treatment, namely, preoperative chemotherapy + surgical treatment, would yield better results in patients with cStage II and III cancer. Therefore, chemoradiotherapy is considered as one of the options in patients who are intolerant to surgery or refuse surgery. It is important to select the appropriate radiation dose, irradiation area, and chemotherapy regimen while considering a treatment strategy, and also consider the salvage treatments for residual and recurrent lesions after chemoradiotherapy (Table 3).

**Table 3:** Summary of prospective clinical studies of chemoradiotherapy

Study name	Histological type studied	Regimen	Radiation dose (Gy)	Complete response rate (%)	Survival (%)
JCOG9708	cStage Ib SCC	Cisplatin 70 mg/m^2^ on days 1 and 36 5-FU 700 mg/m^2^ on days 1–4 and 36–39	60	87.5	4-year survival 80.5
RTOG85-01	cStage I, II, III SCC, AC	Radiotherapy alone	64	NA	5-year survival 0
		Cisplatin 75 mg/m^2^ on days 1 and 29 5-FU 1000 mg/m^2^ on days 1–4 and 29–32	50	NA	5-year survival 27
RTOG94-05	cStage I, II, III SCC, AC	Cisplatin 75 mg/m^2^ on days 1 and 29 5-FU 1000 mg/m^2^ on days 1–4 and 29–32	50.4	NA	2-year survival 40
		Cisplatin 75 mg/m^2^ on days 1 and 29 5-FU 1000 mg/m^2^ on days 1–4 and 29–32	64.8	NA	2-year survival 31
JCOG9906	cStage II, III SCC	Cisplatin 40 mg/m^2^ on days 1, 8, 36 and 43 5-FU 400 mg/m^2^ on days 1–5, 8–12, 36–40, and 43–47	60	62.2	3-year survival 44.7
mRTOG	cStage II, III SCC	Cisplatin 75 mg/m^2^ on days 1 and 29 5-FU 1000 mg/m^2^ on days 1–4 and 29–32	50.4	70.6	3-year survival 63.8
JCOG9516	Unresectable local SCC	Cisplatin 70 mg/m^2^ on days 1 and 36 5-FU 700 mg/m^2^ on days 1–4 and 36–39	60	15	2-year survival 31.5
JCOG0303	Unresectable local SCC	Cisplatin 70 mg/m^2^ on days 1 and 29 5-FU 700 mg/m^2^ on days 1–4 and 29–32	60	0	3-year survival 25.9
		Cisplatin 4 mg/m^2^/5 doses weekly for 6 weeks 5-FU 200 mg/m^2^/5 doses weekly for 6 weeks	60	1.4	3-year survival 25.7
KROSG0101/JROSG021	cStage II, IVA local SCC	Cisplatin 70 mg/m^2^ on days 1 and 29 5-FU 700 mg/m^2^ on days 1–5 and 29–33	60	NA	2-year survival 46
		Cisplatin 7 mg/m^2^ on days 1–5, 8–12, 29–33 and 36–40 5-FU 250 mg/m^2^ on days 1–14 and 29–42	60	NA	2-year survival 44
KDOG0501	Unresectable local SCC	Cisplatin 40 mg/m^2^ on days 1, 15, 29, and 43 5-FU 400 mg/m^2^ on days 1–5, 15–19, 29–33, and 43-47 Docetaxel 20–40 mg/m^2^ on days 1, 15, 29, and 43	61.2	42.1	1-year survival 63.2

*SCC* squamous cell carcinoma, *AC* adenocarcinoma, *5-FU* 5-fluorouracil, *NA* not available

### General remarks

#### Chemoradiotherapy for cStage 0 and I disease

Chemoradiotherapy is indicated for lesions covering ≥ 3/4th of the circumference, which are difficult to treat endoscopically, and those invading up to the submucosa or deeper. The JCOG9708 Study showed good results, with a complete response rate of 87.5% and a 4-year survival rate of 80.5% [26]. Although 9 patients (12.5%) had residual cancer and 30 (41%) developed recurrence after treatment, many of these lesions could be completely cured by endoscopic treatment or surgical resection, and only 9 patients had lesions that could not be radically resected at recurrence. cStage I patients are known to frequently develop recurrent or metachronous multiple lesions in the esophagus after complete response [60], and it is important to perform CT and endoscopy every 3–4 months for at least 2 years after complete response is obtained, and subsequently every 6 months, for detecting recurrent or metachronous multiple lesions at a sufficiently early stage as to allow the lesions to be treated endoscopically.

In addition, it has been reported that 10–50% of patients with obvious submucosal invasion or intramucosal lesions with vascular invasion after endoscopic treatment develops lymph-node metastasis, and these patients were likely to have non-curative resection [61]. For additional treatment of these patients, radical surgery with lymph-node dissection is currently used as the standard of care, while one report has suggested the usefulness of prophylactic chemoradiotherapy in combination with cisplatin + 5-FU for regional lymph nodes [62]. In the JCOG0508 Study, cT1bN0 esophageal cancer with a limited depth of invasion (up to SM2), which was estimated to be treatable endoscopically, was treated endoscopically, and patients with pathologically confirmed complete resection who had pT1a with positive vascular invasion or pT1b received prophylactic chemoradiotherapy. With such treatment, these patients showed a 3-year survival rate (primary endpoint of the study) of 90.7% (90% CI 84.0–94.7). On the other hand, 3 (20%) of the 15 patients who had positive surgical margins after endoscopic treatment and received definitive chemoradiotherapy died of the disease. It should be carefully investigated as to which subpopulation of patients with cT1bN0 disease would be suitable candidates for this treatment. The clinical study was presented at the Annual Meeting of the American Society of Clinical Oncology in June 2016, and its publication is awaited.

#### Chemoradiotherapy for cStage II and III disease

According to one report, chemoradiotherapy was equivalent to surgery alone for cStage II and III cancer [63]. However, according to the JCOG9906 study, chemoradiotherapy was associated with a complete response rate of 62.2%, 3-year survival rate of 44.7%, and 5-year survival rate of 36.8%, which were considered to be inferior results to those of preoperative chemotherapy + surgery in the same subject population (5-year survival rate of 55%, JCOG9907 Study), although no direct comparison can be made. Therefore, chemoradiotherapy is recommended for patients who refuse surgery or are intolerant to surgery, as a treatment with which complete cure can be expected [64]. The RTOG9405/INT0123 study conducted by the US RTOG compared cisplatin (75 mg/m^2^ on days 1 and 29) + 5-FU (1000 mg/m^2^ on days 1–4 and 29–32) chemotherapy with radiotherapy at a radiation dose of 50.4 Gy, and the same chemotherapy with radiotherapy at a radiation dose of 64.8 Gy, and revealed that, while the survival was not prolonged any further, higher toxicity was obtained in the 64.8 Gy group [54]. Based on this, chemotherapy with cisplatin (75 mg/m^2^ on days 1 and 29) + 5-FU (1000 mg/m^2^ on days 1–4 and 29–32) combined with radiotherapy at a radiation dose of 50.4 Gy (RTOG regimen) is considered as one of the standard chemoradiotherapy treatment regimens. A phase II study of a modified RTOG (mRTOG) regimen in Japan reported that addition of prophylactic irradiation of the regional lymph nodes to the original RTOG regimen yielded good results, with a complete response rate of 70.6% and 3-year survival rate of 63.8% [65]. Late toxicity was reduced in the mRTOG regimen when the radiation dose of 50.4 Gy was used, as compared with that in the JCOG9906 study, in which the radiation dose used was 60 Gy. However, attention should be paid to the development of myelosuppression, mucositis, and gastrointestinal symptoms associated with the increased doses of the chemotherapeutic agents. In addition, active salvage treatment, described below, also contributed to the improved treatment outcomes, and it is necessary to consider treatment strategies including salvage treatments after chemoradiotherapy. Criteria for indications of the mRTOG regimen combined with salvage treatment and the safety of salvage treatment are under investigation in the JCOG0909 Study.

#### Chemoradiotherapy for cStage IVa esophageal cancer

When a lesion that is not amenable to surgical resection is limited to the irradiation area, chemoradiotherapy is used as a standard treatment. A single-center phase II study of cisplatin + 5-FU in combination with radiotherapy at a radiation dose of 60 Gy reported a complete response rate of 33% and a 3-year survival rate of 23%, and a multicenter study, the JCOG9516 Study, reported a complete response rate of 15% and a 2-year survival rate of 31.5% [66, 67]. As a result, chemoradiotherapy with cisplatin + 5-FU has come to be used as a standard treatment. Two randomized studies comparing standard chemotherapy with 5-FU (700 mg/m^2^ on days 1–4 and 29–32) + cisplatin (70 mg/m^2^ on days 1 and 29) and low-dose chemotherapy with 5-FU (200 mg/m^2^) + cisplatin (4 mg/m^2^) on days 1–5, 8–12, 15–19, 22–26, 29–33, and 36–40, both combined with radiation at the dose of 60 Gy, failed to find any clear advantage of the low-dose chemotherapy [68, 69]. A clinical study of DCF therapy, in which docetaxel is added to cisplatin + 5-FU, in combination with radiotherapy reported good results with a complete response rate of 42.1%; however, Grade 3–4 esophagitis or febrile neutropenia occurred in ≥ 30% of the subjects. Therefore, adoption of this treatment needs to be carefully considered [70]. Multidisciplinary treatment in which surgery or chemoradiotherapy is performed after intensive induction chemotherapy has been shown to yield good short-term results with a 1-year survival rate of 67.9% [71], and a comparative study (JCOG1510) is planned.

#### Radiation dose and chemotherapy regimens used in chemoradiotherapy

The RTOG8501 study recommended chemoradiotherapy as a standard treatment, because comparison of radiotherapy (64 Gy) alone and concurrent chemoradiotherapy (cisplatin + 5-FU + 50 Gy) for esophageal cancer revealed significantly superior treatment outcomes of chemoradiotherapy [72]. In addition, a meta-analysis of studies of chemotherapy and radiotherapy reported that concurrent chemotherapy and radiotherapy yielded a significantly greater prolongation of the survival than sequential chemotherapy and radiotherapy [73]. Furthermore, the above-mentioned RTOG9405/INT0123 study revealed no superior outcomes in terms of the survival or local control rate in the high-dose group, concluding that a radiation dose of 50.4 Gy should be used in combination with cisplatin (75 mg/m^2^ on days 1 and 29) + 5-FU (1000 mg/m^2^ on days 1–4 and 29–32) chemotherapy. Many studies in Japan have used a radiation dose of 60 Gy in combination with lower doses of the antitumor agents, such as cisplatin (70 mg/m^2^ on days 1 and 29) and 5-FU (700 mg/m^2^ on days 1–4 and 29–32) [74, 75]. For multidisciplinary treatment including salvage treatment, the mRTOG regimen has also been increasingly used, and its usefulness is now under investigation in the JCOG0909 Study.

#### Adverse effects of radical chemoradiotherapy

Adverse effects of chemoradiotherapy are mainly classified into acute and late toxicity. Acute toxicity occurs mainly during concurrent chemotherapy and radiotherapy, within 1–2 months after the start of treatment. Late toxicity is often associated with radiation and occurs a few months to a few years after completion of treatment. Symptoms of acute toxicity include gastrointestinal toxicity, nausea, vomiting, renal impairment, leukopenia, esophagitis, and dysphagia, and should be treated according to guidelines such as the “Guidelines for Proper Use of Antiemetics” and “Practical Guideline of Febrile Neutropenia (FN)”. Symptoms of late toxicity include radiation pneumonitis, pleural effusion, pericardial effusion, pericarditis constrictive, and hypothyroidism, which interfere with daily life in approximately 10% of patients [76–78]. Since late toxicity may be lethal, regular follow-up, medical interviews to obtain information on subjective symptoms such as dyspnea, and early treatment are important.

#### Salvage treatment for local residual/recurrent lesions after radical chemoradiotherapy

When there is a local residual or recurrent lesion after chemoradiotherapy for esophageal cancer, salvage surgery or endoscopic treatment may allow long-term survival. It has been reported that, in salvage surgery, R0 resection allows long-term survival, but, at the same time, increases the incidence of postoperative complications and in-hospital mortality [79–83]. When a residual lesion remains confined in the mucosa, salvage endoscopic treatment can be performed safely [84, 85]. Photodynamic therapy (PDT) has been reported to yield good results even in cases with suspected invasion of the submucosa or muscularis propria, and PDT is considered as one of the potentially useful treatment options [86].

## Follow-up after treatment of esophageal cancer

### Summary

The purpose of follow-up after treatment of esophageal cancer is (1) to detect and treat recurrence early, and (2) to detect and treat multiple/double cancers early. Furthermore, follow-up is also important from the standpoint of systemic management and knowing the QOL of the patients after treatment.

Methods of follow-up after treatment of esophageal cancer vary depending on the type of initial treatment and on the stage of cancer progression at the time of the initial treatment. During follow-up, it is important to keep in mind that the early detection/treatment may allow long-term survival and to pay attention to the occurrence of metachronous multiple esophageal cancers and metachronous double cancers in other organs, mainly high-incidence cancers, i.e., gastric cancer and head and neck cancer. Establishment of a consensus-based follow-up system and verification of its effectiveness are required.

### General remarks

#### Follow-up after endoscopic resection

No certain method of follow-up after endoscopic resection has been established. Local recurrence often occurs within 1 year after the initial treatment, although it, sometimes, takes up to 2–3 years after the initial treatment, and long-term-follow-up is required [87, 88]. Esophagoscopy with iodine staining is mainly used to screen for local recurrence, and many studies have reported that screening for local recurrence is performed every 3 or 6 months for 1 year after resection [3, 4, 87–89]. Patients with piecemeal resection and those with multiple iodine-unstained areas have a high risk of local recurrence, requiring a more strict esophagoscopy protocol [3, 4, 87, 88, 90]. Lymph-node recurrence/organ recurrence may be detected 2–3 years later, and regular, long-term follow-up is required [5, 91].

In regard to the methods of examination, follow-up is usually performed every 6–12 months using several equipments such as contrast-enhanced thoracoabdominal CT and EUS [92]. For example, in the JCOG0508 Study “Single-arm confirmatory study on efficacy of combined treatment of endoscopic mucosal resection and chemoradiotherapy for clinical stage I esophageal carcinoma,” medical examinations and contrast-enhanced neck to abdominal CT and measurement of squamous cell carcinoma (SCC) antigen, a tumor marker, are to be performed every 4 months for 3 years after EMR.

#### Follow-up after radical surgery

Recurrence after radical surgery occurs in 29–43% of cases in Japan. Although, in approximately 85% of cases, the recurrences occur early, often within 2 years after surgery, in some cases, they occur much later, and this should be borne in mind [93–95]. The patterns of recurrence include lymph-node recurrence, local recurrence, organ recurrence and disseminated recurrence, and mixed type of recurrence [95].

The actual method of follow-up after radical resection of esophageal cancer is currently determined by each institution, and no studies have clarified the usefulness of regular follow-up or an effective method of follow-up. A nationwide survey conducted by the Guideline Committee [96] revealed that many institutions perform follow-up with tumor markers and diagnostic imaging, mainly CT, ≥ 4 times a year during the first 2 years after resection and at least twice a year from the third year until the fifth year, and that some institutions perform follow-up for up to 10 years. Mainly contrast-enhanced thoracoabdominal CT and upper gastrointestinal endoscopy are performed as follow-up examinations, and neck/abdominal US, bone scintigraphy and PET-CT are performed as necessary. CT is performed every 3–6 months in many institutions, and the frequency of CT often varies depending on the stage of cancer progression and on the number of years after surgery.

#### Follow-up after radical chemoradiotherapy

CT, esophagoscopy, and other examinations are usually used for follow-up after radical chemoradiotherapy, but there are no reports clarifying the optimal frequency of these examinations or the optimal follow-up period. According to a nationwide survey [96], follow-up is performed every 3 months during the first year after chemoradiotherapy at most institutions. For patients with cStage II or more advanced cancers, follow-up similar to that in the first year is performed up to the third year at many institutions, and follow-up is continued for at least 5 years after treatment at all the institutions surveyed. Primary esophageal lesions and lymph-node metastasis are commonly encountered as residual/recurrent lesions after chemoradiotherapy, and in most of these cases, these are detected within 1 to 2 years after the start of treatment.

After definitive chemoradiotherapy for esophageal cancer, not only screening for detecting recurrence, but also follow-up for the early identification of the late effects of radiotherapy such as radiation pneumonitis, pleural effusion, and pericardial effusion is necessary [76]. These late effects may greatly impair the patients’ QOL, and patients could die of the late effects.

#### Points to consider in patients with metachronous multiple esophageal cancers and double cancers in other organs

Esophageal cancer is characterized by relatively frequent occurrence, metachronously, of multiple cancers in the esophagus. In addition, development of metachronous cancer in other organs, such as gastric cancer and head and neck cancer, is also not rare [97, 98]. Bearing this in mind, upper gastrointestinal endoscopy needs to be regularly performed to carefully observe the pharynx, entire esophagus (remnant esophagus in surgical cases), and stomach. Particular attention needs to be paid to the development of metachronous head and neck cancer in patients with multiple iodine-unstained areas and those with head and neck cancer [98, 99]. Magnifying endoscopy with narrow-band imaging (NBI) is useful for detecting superficial head and neck cancer [100]. Furthermore, attention also needs to be paid to the development of colorectal and other cancers.

## Treatment of recurrent esophageal cancer

### Summary

Since there are a variety of initial treatments for esophageal cancer, such as endoscopic treatment, radical surgery, and definitive chemoradiotherapy, treatment for recurrent esophageal cancer needs to be considered individually according to the type of initial treatment. Furthermore, treatment varies depending on whether the pattern of recurrence is lymph-node recurrence, local recurrence, distant organ recurrence, or mixed recurrence, and the general condition of the patient at the time of recurrence also affects the choice of treatment. It is difficult to conduct large-scale clinical studies on the treatment of recurrent esophageal cancer, and there is currently little evidence of the usefulness of any type of treatment used. While cure may be achieved depending on the type of recurrence, for example, by salvage therapy after radical chemoradiotherapy, treatment for suppressing tumor exacerbation or improving QOL is also often used.

### General remarks

#### Treatment of recurrence after endoscopic resection

Local recurrence after endoscopic mucosal resection often develops within 1 year after the initial treatment, but may sometimes take until 2 to 3 years after the initial treatment. Recently, indications for endoscopic resection have been expanded in clinical studies. There are no certain indications for, or evidence for the type of additional treatment after endoscopic resection, and some patients receive follow-up alone (see Chapter “Endoscopic treatment”).

#### Treatment of recurrence after radical surgery

Recurrence after radical surgery for esophageal cancer has been reported to occur in 28–47% of cases in Japan [93, 101], while the reported recurrence rates of ≥ 50% are not rare in reports from Europe and North America [102, 103]. In regard to the patterns of recurrence, 22–68% of cases show lymph node/local recurrence, 12–51% show distant organ metastasis, and 7–27% show a mixture of both types of recurrence. Recurrence in the neck/superior mediastinum is common in cases of lymph-node recurrence, while, in cases of distant organ metastasis, the lung is the most common site of recurrence, followed in frequency by the liver, bone, and brain. Even metastases to the small intestine and colon have been reported.

Patients with recurrence after radical resection for esophageal cancer have extremely poor survival rates, with a median survival duration of 5–10 months after diagnosis. On the other hand, cases of long-term survival and those of complete cure have also been reported, and active treatment for recurrent lesions should be considered [93, 101–113].

Treatment of recurrence after radical resection for esophageal cancer is selected according to the site, pattern, and extent of recurrence. Treatment varies depending on the condition in each individual, such as the general condition of the patient at the time of recurrence, whether recurrence is in the surgical area, and whether irradiation was given preoperatively or postoperatively. Therefore, there have been a few reports of large-scale studies of the treatment outcomes according to various pathological conditions.

#### Treatment of recurrence developing after complete response to definitive chemoradiotherapy

In recent years, definitive chemoradiotherapy has been increasingly chosen as the initial treatment, not only for unresectable esophageal cancer, but also for cases with esophageal cancer that is judged as being resectable. Although complete response has been achieved in many cases, recurrences, including local recurrence, are often encountered. The treatment of recurrence varies depending on the pathology and general condition of the patient, and no consensus has been reached. However, when the recurrence is localized, salvage treatment such as surgery and endoscopic resection may be adopted [79, 84, 85, 105, 114–117] (see chapter “Multidisciplinary treatment”, Chemoradiotherapy).

## Palliative care

### Summary

While palliative care should be commonly provided for cancers at any site, in esophageal cancer patients, dysphagia, malnutrition, cough due to fistula formation with the airways, and other symptoms often decrease the QOL, and provision of treatment for relieving these symptoms and maintaining/improving the QOL of the patients should be considered from even the early stages of treatment. However, the method of palliation adopted is determined by the prevailing practice at individual institutions, and further evaluation is required. All medical professionals need to master the knowledge and skills needed in palliative care.

### General remarks

The World Health Organization (WHO) (2002) defines palliative care as “an approach that improves the quality of life of patients and their families facing problems associated with life-threatening illness, through the prevention and relief of suffering by means of early identification and impeccable assessment and treatment of pain and other problems, physical, psychosocial, and spiritual.” The Second Basic Plan to Promote Cancer Control Programs in fiscal year 2012 states that “promotion of palliative care from the time of cancer diagnosis” is an issue that needs to be focused on. The above-mentioned palliative care is common to all cancer patients and provided in daily practice, not only the attending physicians and nurses, but also palliative care specialists, psycho-oncologists, clinical psychologists, dentists, pharmacists, certified social workers, physical therapists, and other professionals need to engage and provide team care. Methods based on the “Guidelines for Pharmacotherapy of Cancer Pain” established by the Japanese Society for Palliative Medicine are recommended for cancer pain.

Patients with esophageal cancer often suffer from dysphagia and malnutrition due to esophageal obstruction, cough due to aspiration/fistula, and chest pain due to the tumor, resulting in a lowered QOL already at the time of diagnosis. Even while providing treatment for cure, it is important, from the early stage, to provide treatment for the relief of symptoms and for maintaining/improving the QOL of the patients [118].

In palliative care for patients with terminal esophageal cancer, problems that need to be handled are, in particular, dysphagia due to esophageal obstruction and malnutrition caused by dysphagia, symptoms caused by airway obstruction or fistula formation with the airway, cachexia, and other symptoms due to distant metastases and hypercalcemia. To improve the symptoms of esophageal obstruction and airway obstruction and those caused by fistula, palliative radiotherapy, chemoradiotherapy, esophageal stenting, airway stenting, esophageal bypass surgery, and/or other treatments may be used [119, 120] (see Chapter “Radiotherapy”; Chapter “Multidisciplinary Treatment”, Chemoradiotherapy).

For improving dysphagia in unresectable esophageal cancer, a Cochrane Database Systematic Review in 2014 showed that self-expandable esophageal metallic stents are more effective and faster-acting than plastic stents and other methods [121]. However, it should be kept in mind that stenting may also cause complications, causing pain and further decreasing the QOL, and the treatment(s) should be undertaken after providing adequate explanation to the patient and obtaining informed consent. In addition to esophageal stenting, intracavitary irradiation, laser irradiation, hyperthermia, ethanol injection, etc., have been reported as treatments for providing relief from esophageal obstruction. While intracavitary irradiation may act more slowly in providing relief from esophageal obstruction than esophageal stenting, it could be a useful alternative treatment to esophageal stenting, as it is associated with a lower incidence of complications, provides more sustained relief from esophageal obstruction, and may be expected to prolong the survival and improve the QOL [121]. However, intracavitary irradiation alone is scarcely adopted as a treatment option in Japan (see Chapter “Radiotherapy”).

Patients with tracheoesophageal fistula formation have a reduced QOL due to repeated episodes of aspiration and pneumonia, but placement of a covered self-expandable esophageal stent, and in some cases, placement of an airway stent in addition to the esophageal stent, have been reported to be effective [122].

In patients with severe obstruction who have already undergone definitive chemoradiotherapy or radiotherapy and in whom radical resection cannot be expected, if it is considered that esophageal stenting would be difficult or dangerous, a nutritional fistula may be created to allow the patient to be switched to home care. Percutaneous endoscopic gastrostomy, which can usually be performed using an endoscope, is effective, and may be performed even before the start of the multidisciplinary treatments in patients with severe obstruction [123]. In cases in which percutaneous endoscopic gastrostomy is difficult due to severe obstruction, such that even a small-diameter endoscope is difficult to negotiate through, or in patients with a history of abdominal surgery, open gastrostomy or jejunostomy may be performed.

In addition, medical professionals involved in the treatment of esophageal cancer often encounter potentially fatal complications, such as sudden respiratory arrest due to airway obstruction and massive hematemesis due to aortic perforation. Since it is difficult to save the lives in most of these cases, it is important to provide a thorough explanation in advance, particularly to the patients’ families. Patients and their families are thus often forced to live in fear of sudden change/death, and psychological support and mental care for them are indispensable.

## Diagnosis and treatment of Barrett’s esophagus and Barrett’s carcinoma

### Summary

An esophagus lined with Barrett’s mucosa is called Barrett’s esophagus [124]. Barrett’s mucosa is endoscopically recognizable columnar epithelium extending from the stomach to the esophagus and does not require histological confirmation of specific columnar epithelial metaplasia [125–129]. Identification of the esophagogastric junction is required for the diagnosis of Barrett’s mucosa, and the endoscopically identifiable distal end of the lower esophageal palisade vessels is defined, in principle, as the esophagogastric junction. Barrett’s mucosa is characterized by at least one of the following histological findings: (1) esophageal gland ducts in the mucosa beneath the columnar epithelium or esophageal glands proper in the submucosa; (2) squamous islands within the columnar epithelium; (3) double muscularis mucosae beneath the columnar epithelium. Barrett’s carcinoma is defined as adenocarcinoma arising from Barrett’s mucosa. Early, superficial, and advanced cancer are defined in the same manner as for the case of esophageal squamous cell carcinoma, in general, but the deep muscularis mucosae is handled as the genuine muscularis mucosae. Barrett’s carcinoma is treated in accordance with the treatment principles for esophageal squamous cell carcinoma at the cancer site. Endoscopic resection is currently indicated for lesions extending in depth down to the lamina propria (EP: remaining in the epithelium, non-invasive lesion; SMM [superficial muscularis mucosae]: remaining in the superficial muscularis mucosae; LPM [lamina propria mucosae]: not reaching the deep muscularis mucosae); however, accumulation of cases is necessary for establishing the optimal treatment.
